# Supplementary material for: The adapted French version of the Academic and Athletic Identity Scale (AAIS-FR): Evidence of validity and reliability and relationships with sport well-being
Source: PLoS One. 2024 May 8;19(5):e0298872. doi: 10.1371/journal.pone.0298872 (PMC11078428; doi:10.1371/journal.pone.0298872)
Supplement: S1 Checklist — (DOCX) [file pone.0298872.s001.docx]

**Human Participants Research Checklist**

***Complete the following if your study involved human participants or human participants’ data. These questions should be addressed for prospective and retrospective studies.***

1. Did you obtain ethics approval for this study?
   - If yes, please upload (file type “Other”) the original approval document you received from your ethics committee. If the original document is in another language, please also provide an English translation.

___ Uploaded _X__ N/A

- - If you did not obtain ethical approval, please explain why this was not required below.

In France, the need for ethical approval depends on the type of study being conducted and the type of data being collected. A study that is completely anonymous (i.e., without any means of identifying the respondent, such as address, date of birth, etc.), conducted online, and does not involve sensitive questions about the health of individuals (as is the case here) does not require ethical approval. However, researchers must comply with the regulations of the French Data Protection Agency, the “Commission Nationale de l'Informatique et des Libertés” (CNIL, https://www.atinternet.com/en/glossary/cnil/), which regulates the collection and processing of personal data. Prior to the start of the study, CNIL approval was obtained from the data protection officer at the first author's university.

1. If you prospectively recruited human participants for the study – for example, you conducted a clinical trial, distributed questionnaires, or obtained tissues, data or samples for the purposes of this study, please report in the Methods:
   1. the day, month and year of the **start and end** of the recruitment period for this study.
   2. whether participants provided informed consent, and if so, what type was obtained (for instance, written or verbal, and if verbal, how it was documented and witnessed). If your study included minors, state whether you obtained consent from parents or guardians. If the need for consent was waived by the ethics committee, please include this information.

_X__ Completed ___ N/A

1. If you are reporting a retrospective study of medical records or archived samples, please report in the Methods section:
2. the day, month and year when the data were accessed for research purposes
3. whether authors had access to information that could identify individual participants during or after data collection

___ Completed _X__ N/A
