# Supplementary material for: The adapted French version of the Academic and Athletic Identity Scale (AAIS-FR): Evidence of validity and reliability and relationships with sport well-being
Source: PLoS One. 2024 May 8;19(5):e0298872. doi: 10.1371/journal.pone.0298872 (PMC11078428; doi:10.1371/journal.pone.0298872)
Supplement: S1 Appendix — (DOCX) [file pone.0298872.s003.docx]

**The French version of the AAIS (AAIS-FR).**

Imaginez que la figure suivante est un diagramme qui vous représente. Chacun des cercles concentriques représente les caractéristiques qui sont centrales à votre sentiment d’identité en tant que personne.

S’il vous plait pensez à la figure en évaluant les items ci-dessous.

La plupart des gens vont utiliser différentes réponses, en estimant que certaines qualités sont très importantes/centrales et que d’autres ne sont pas essentielles/centrales à leur perception d’eux-mêmes. Pour avoir une bonne idée de la façon dont vous allez comparer et évaluer les différentes qualités, veuillez lire l’ensemble des items avant de commencer à évaluer chacun d’entre eux.


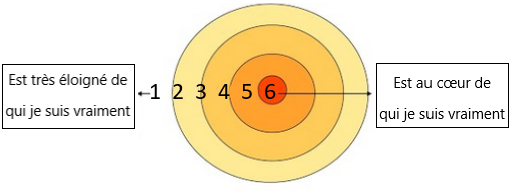


Indiquez, s’il vous plait, dans quelle mesure chacune de ces caractéristiques ci-dessous est essentielle à la perception que vous avez de **votre identité**. Si une qualité vous semble bonne ou souhaitable, mais qu'elle ne constitue pas une part importante de votre identité, alors vous devez répondre « pas du tout au cœur de mon identité » (1). Entourez la réponse qui correspond le mieux à votre opinion concernant la place centrale de chaque caractéristique dans votre identité.

| *Dans quelle mesure chacune de ces caractéristiques ci-dessous est essentielle à la perception de votre identité* | Est très éloigné de qui je suis vraiment |  |  |  |  | Est vraiment au cœur de qui je suis vraiment |
| --- | --- | --- | --- | --- | --- | --- |
| 1. Être un·e étudiant·e compétent·e | 1 | 2 | 3 | 4 | 5 | 6 |
| 2. Être satisfait·e de mon travail universitaire. | 1 | 2 | 3 | 4 | 5 | 6 |
| 3. Bien réussir à l’université. | 1 | 2 | 3 | 4 | 5 | 6 |
| 4. Avoir de bonnes notes. | 1 | 2 | 3 | 4 | 5 | 6 |
| 5. Faire parti·e des meilleur·es étudiant·es. | 1 | 2 | 3 | 4 | 5 | 6 |
| 6. Être un·e athlète compétent·e | 1 | 2 | 3 | 4 | 5 | 6 |
| 7. Être un·e bon·ne athlète*.* | 1 | 2 | 3 | 4 | 5 | 6 |
| 8. Être sportif·ve | 1 | 2 | 3 | 4 | 5 | 6 |
| 9. Être satisfait·e de mes performances sportives | 1 | 2 | 3 | 4 | 5 | 6 |
| 10. Bien réussir lors des compétitions sportives*.* | 1 | 2 | 3 | 4 | 5 | 6 |
